# Landscape Grain Effect in Yancheng Coastal Wetland and Its Response to Landscape Changes

**DOI:** 10.3390/ijerph16122225

**Published:** 2019-06-24

**Authors:** Peng Tian, Luodan Cao, Jialin Li, Ruiliang Pu, Xiaoli Shi, Lijia Wang, Ruiqing Liu, Hao Xu, Chen Tong, Zijing Zhou, Shuyao Shao

**Affiliations:** 1 Donghai Institute, Ningbo University, Ningbo 315211, China; tppyang@163.com; 2 Department of Geography & Spatial Information Techniques, Ningbo University, Ningbo 315211, China; ljwang2013@163.com (L.W.); liuruiqingwh@163.com (R.L.); xuhao@nbu.edu.cn (H.X.); gdyff0@163.com (C.T.); tomatozzj@outlook.com (Z.Z.); vickyssy@163.com (S.S.); 3 School of Geosciences, University of South Florida, Tampa, FL 33620, USA; rpu@usf.edu; 4 Editorial Department of Journal of Ningbo University, Ningbo 315211, China; shixiaoli@nbu.edu.cn

**Keywords:** grain effect, landscape pattern, landscape level, class level, coastal wetland, Yancheng

## Abstract

The landscape grain effect reflects the spatial heterogeneity of a landscape and it is used as a research core of landscape ecology. The landscape grain effect can be used to not only explore spatiotemporal variation characteristics of a landscape pattern, but also to disclose variation laws of ecological structures and functions of landscapes. In this study, the sensitivity of landscape pattern indexes to grain sizes 50–1000 m was studied based on landscape data in Yancheng Coastal Wetland acquired in 1991, 2000, 2008, and 2017. Response of the grain effect to landscape changes was analyzed and an optimal grain size for analysis in the study area was determined. Results indicated that: (1) among 27 indexes (12 in a class level and 15 in a landscape level), eight indexes were highly sensitive to grains, ten indexes presented moderate sensitivity, eight indexes presented low sensitivity, and one was unresponsive. It was shown that the area-margin index and the shape index were more sensitive to the different grain sizes. The aggregation index had some differences in the grain size change, and the diversity index had a low response degree to the grain size. (2) Landscape indexes showed six different responses to different grains, including slow reduced response, fast reduced and then slow reduced response, monotonically increased response, fluctuating reduced response, up-down responses, and stable response, which indicated that the landscape index was closely related to the spatial grain. (3) From 1991 to 2017, variation curves of the landscape grain size of different landscape types could be divided into four types: fluctuation rising type, fluctuation type, monotonous decreasing type, and monotonous rising type. Different grain size curves had different interpretations of landscape changes, but in general, Yancheng Coastal Wetland’s landscape tended to be fragmented and complicated, internal connectivity was weakened, and dominant landscape area was reduced. Natural wetlands were more sensitive to grain size effects than artificial wetlands. (4) The landscape index at the 50 m grain size had a strong response to different grain size changes, and the loss of landscape information was the smallest. Therefore, it was determined that the optimal landscape grain size in the study area was 50 m.

## 1. Introduction

Since the birth of the term “landscape ecology” in 1939, this discipline has been developed rapidly in theory, methods, and applications [1,2,3]. Landscape ecologists in the world have held two conferences associated with special discussion on key issues and priority research areas of landscape ecology in 2001 and 2003. Professor Wu and Professor Richard Hobbs [4,5] summarized the contents of the conferences as the “Top Ten Research Topics in Landscape Ecology”. Among them, scaling is one of the most important topics in the research and practice of landscape ecology theory [6,7]. The scaling mainly refers to translating information from one scale to another, which inevitably involves multiscale and spatial heterogeneity [8,9,10]. Spatial heterogeneity mainly refers to the complexity of spatial distribution pattern of landscape structure and functional, which widely exists at multiple scales and is scale-dependent [11]. It shows that the measurement of spatial heterogeneity or landscape pattern is closely related to a selected observation or analysis scale. Since the 21st century, the issue of scale and scaling has become a very important and cutting-edge topic in the study of ecology [8,12,13,14]. It involves all aspects of ecology in theory, method, and practice [15,16,17].

Size is a basis to study landscape size and it reflects heterogeneity and differences in landscape space [18]. Analyzing evolution characteristics of landscape under different scale effects is a key research direction and a basic problem of current landscape ecology [19,20,21]. Scale includes temporal and spatial grain and extent [22,23]. Temporal grain reflects the interval or frequency of a change, while spatial grain reflects units or pixels with different resolutions. Extent refers to an area of a region [24,25]. Research on the scale effect refers to the spatial effect analysis of different grain sizes, which is the spatial grain effect of landscape pattern. The structure, morphology, information, and function of a landscape are closely related to the grain size [26,27]. Landscapes at a larger scale require analysis of the ecological effects of landscape at different scales, which is conducive to guaranteeing the integrity and accuracy of landscape information [28,29]. Landscape ecologists have noticed this phenomenon for a lone time [30,31,32]. Turner et al. [30] analyzed patch type diversity, dominance, and contagion response to spatial amplitude changes. Saura and Martinez [31] analyzed the sensitivity of eight landscape indices to amplitude changes. Wu et al. [32] analyzed the amplitude effects of various landscape indices using four kinds of real landscapes, and found that the calculation results of landscape pattern indices varied with spatial extent. Studying the characteristics of landscape pattern under different grains has important theoretical and practical significance for efficient utilization of landscape resources, rational analysis on the ecological processes of landscapes, and the improvement of research accuracy.

The scale effect is a key research concept of landscape ecology [19,20,21]. Currently, there are many studies on scale effect of landscape pattern [33,34]. Study data were collected from interpretations of remote sensing satellites and land use data [35,36,37]. For example, Lü et al. [35] compared the resampling method with high-resolution data and the multisource and multiresolution data (MSMRD) method, and carried out a grain analysis of a small watershed landscape on a loess plateau. With respect to grain selection, most studies chose the same grain or a grain with an equal growth interval, ranging from 1 to 3000 m. For example, Wu et al. [33] used 10 m, 20 m, and 30 m at different intervals, and selected 35 kinds of grain sizes from 50 to 630 m. The response behaviors of 19 common landscape indices to amplitude changes were analyzed. Wang et al. [38] chose three grains, including 3 × 3 m, 35 × 35 m, and 60 × 60 m. Ren et al. [39] chosen 15 grain units, including 5 m, 10 m, 20 m, 30 m, 40 m, 50 m, 60 m, 70 m, 80 m, 90 m, 100 m, 200 m, 300 m, 400 m, and 500 m. Zhang et al. [40] obtained grid elements of different scales of 20 m, 40 m, 60 m, 80 m, ..., 160 m, 180 m, and 200 m at intervals of 20 m, and a landscape area loss index was combined to determine the particle size value suitable for the research area. The study areas for analyzing the grain effect mainly include on urban, river basin, and ecological preservation areas [41,42]. The research period is usually concentrated in a certain year, and most studies are static research on the relationship between landscape pattern and grain [41,42,43]. A breakpoint of the grain curve is subjectively selected as the optimal grain size, which also has certain impacts on the complete and accurate analysis of landscape patterns [43]. The landscape pattern is sensitive to spatial heterogeneity. This means that landscape pattern characteristics differ significantly with different grain sizes. Therefore, studying landscape pattern characteristics under different grains and dynamic analysis of responses of the grain effect to landscape changes at different time points are of important significance. Currently, there are universal static studies on grain effects [44,45]; dynamic studies on grain effects at different time points can supplement such studies to some extent. Moreover, a combination of dynamic studies at different scales with landscape area loss index can help to more objectively select an optimal grain for analysis.

The Yancheng Coastal Wetland landscape has important ecological characteristics of coastal wetland landscapes in China. Determining the granularity of the Yancheng Coastal Wetland landscape is of great significance for safeguarding regional ecology and promoting environmental protection measures based on local conditions [46]. With a rapid increase in the intensity of marine development in Jiangsu Province, China, the scale of port development, tidal flat reclamation, and fish pond breeding has been expanded. Therefore, the Yancheng Coastal Wetland ecosystem’s stability is under threat of degradation of regional biodiversity and ecological function [47]. As an important wetland preservation area in China and the world, Yancheng Coastal Wetland has been widely studied. Many studies on the landscape ecology in Yancheng Coastal Wetland have been documented. These studies cover a long study period and focus on expansion and shrinkage, ecological service functions, influences of sea reclamation, and introduction and protection of animals and plants in the coastal wetland [48,49,50,51]. Zhang et al. [48] analyzed area changes in Yancheng Coastal Wetland since the 1960s and the relative driving forces. Kang et al. [49] and Li et al. [50] studied the development of Yancheng Coastal Wetland under the influence of sea reclamation. However, there are few studies on grain effect of landscape patterning in Yancheng Coastal Wetland. Sun and Liu [51] carried out simple studies in Yancheng Coastal Wetland, and concluded that an appropriate scale was 200 m from the perspective of static analysis. Therefore, the existing research lacks dynamic analysis of the grain size effect on the Yancheng Coastal Wetland landscape, and there is a shortage of studies on the grain size variation of multiple landscape pattern indices at different scales.

It is urgent to understand the dynamic changes of the Yancheng Coastal Wetland landscape under the influence of human activities. Because there is a significant scale effect of the landscape cover change, the results of different scales on the same area are very different. Hence, given the grain size effect of a landscape pattern, it is particularly important to deeply explore the response of the grain size effect to the landscape change process. Consequently, it is necessary to determine the optimal spatial grain size of a regional landscape before characterizing the change of the landscape pattern. Thus, in this study, there are four specific objectives: (1) Analyzing the grain effect of Yancheng Coastal Wetland landscape pattern by using both landscape level and class level under the landscape pattern; (2) determining how landscape scale varies with the size of the grain, and how grain size affects the landscape pattern; (3) exploring whether a specific grain size has a more significant effect on the Yancheng Coastal Wetland landscape pattern and natural and artificial wetland landscape changes than another; and (4) selecting the most appropriate grain size to analyze changes of landscape pattern in Yancheng Coastal Wetland.

## 2. Materials and Methods

### 2.1. Study Area

**Figure 1:**
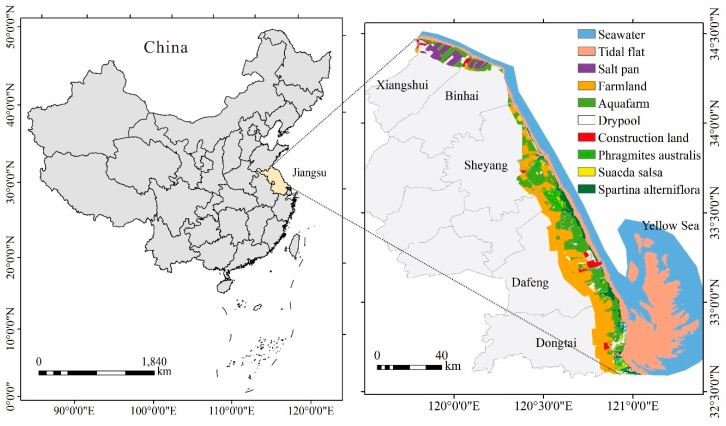
Geographic location of the study area (**left**) and distribution (**right**) of landscape types in 2017.

Yancheng Coastal Wetland is located in 119°27′–121°16′ E and 32°34′–34°28′ N with a shoreline about 530 km long, which is in middle of Jiangsu Province in eastern China, close to the Yellow Sea (Figure 1). The terrain is high in the west and low in the east. Many rivers flow into the Yellow Sea from the west to the east. There is a typical monsoon climate in the study area. The extent of Yancheng Coastal Wetland was determined by referring to the studies of Sun et al. [51] and Kang et al. [49]. The sea boundary is located at the tidal flat edge using remote sensing images acquired in 2017, which covered the sea boundaries in all years to the maximum extent. Yancheng’s main road and river were used as land boundaries. The area enclosed by the sea boundaries and land boundaries was used as the study area. Yancheng Coastal Wetland is not only a world-famous wetland, but is also an important wetland preservation area in China. Yancheng Coastal Wetland possesses extensive natural wetland species, such as *Phragmites australis*, *Suaeda salsa*, and *Spartina alterniflora*. It is a center for reproduction of various rare animals and plants, such as *Grus japonensis* and *Elaphursu davidianus*, etc. There is a long coastline and a wide muddy plain, accompanied by dense distribution of rivers, extensive distribution of wetlands, and extremely high biodiversity.

### 2.2. Data Source and Preprocessing

The Landsat TM/OLI image data used in this study were mainly provided by the website of United States Geological Survey (USGS) (http://glovis.usgs.gov/) and geographical spatial data. The average spatial resolution of the image data was 30 m. Images acquired over four years were chosen, including 1991, 2000, 2008, and 2017. The information of satellite orbital parameters is listed in Table 1. Distribution of landscape types in the study area in the four different years was gained by interpretation of remote-sensing data and field survey. Visual interpretation and human–computer interaction interpretation of remote sensing images were carried out, and the Kappa index for mapping the landscape types in the four different years was above 0.87, indicating that mapping data were qualified and applicable for further processing. Considering the actual distributions of landscape in Yancheng and the demands of research, landscapes in the study area were divided into natural wetland landscape and artificial wetland landscape. Natural wetland landscape covers seawater, tidal flat, *Phragmites australis*, *Suaeda salsa*, and *Spartina alterniflora*. The artificial wetland landscape covers salt pan, farmland, aquafarm, dry pond, and construction lands [47].

### 2.3. Research Methods

#### 2.3.1. Selection and Calculation of Landscape Pattern Indices

In this study, sixteen common landscape indexes (Table 2), including 12 class-level indexes and 15 landscape-level indexes, were chosen for analyzing change characteristics of area-margin, shape, aggregation, and diversity of landscape pattern in the study area. Changes of landscape indexes with grain size were studied. Landscape indexes were mainly divided into patch level, class level, and landscape level. The patch-level indexes are the most basic component of a landscape. Each landscape is composed of patches with different sizes and it reflects characteristics of individual patches, such as area and perimeter. Class-level indexes reflect characteristics of one type of patch and structural changes of a landscape in a region. The landscape-level indexes reflect landscape changes of the all patches in the study area and they reflect integral changes. Both class-level indexes and landscape-level indexes can reflect landscape characteristics in the study area, such as area, morphology, diversity, and fragmentation of one type of landscape or total patches [47]. These indexes were calculated by Fragstats 4.2 (UMass Landscape Ecology Lab, Amherst, MA, USA). The 16 landscape pattern indexes are listed in Table 2.

#### 2.3.2. Spatial Grain Analysis

Selection of spatial grain size is the basic content of grain effect analysis. In this study, landscape grains were selected through small-to-large scales. Previous studies on spatial grain sizes mainly select grains of 1–3000 m due to differences in study area as well as the natural and economic level of the area [38,39]. Large-scale landscape changes might ignore small-scale landscape change characteristics, while small-scale landscape changes can reflect detailed landscape change rules of the large scale [39]. Hence, spatial grain size range in the present study was chosen from 50 × 50 m to 1000 × 1000 m at intervals of 50 m (size of grid pixel = 50 × 50 m). In the study area, twenty spatial grain (grid pixel sizes) data were produced by using the resampling module in ArcGIS 10.5 (Esri Inc., Redlands, CA, USA) spatial analysis, which were 50 m, 100 m, 150 m, 200 m, 250 m, 300 m, 350 m, 400 m, 450 m, 500 m, 550 m, 600 m, 650 m, 700 m, 750 m, 800 m, 850 m, 900 m, 950 m, and 1000 m, respectively.

#### 2.3.3. Responses of Landscape Indexes to Spatial Grains

The coefficient of variation (CV) of landscape index was obtained by calculating the ratio between the standard deviation and the mean in the study area. CV was used to reflect responses and sensitivity of different landscape indexes to grains. With reference to previous studies [45,52], response sensitivity was divided into five levels according to different numerical values of CV, namely, response insensitivity (CV ≤ 1%), weak response sensitivity (1% < CV ≤ 10%), moderate response sensitivity (10% < CV ≤ 50%), strong response sensitivity (50% < CV ≤ 100%), and extremely strong response sensitivity (CV > 100%).

According to landscape indexes under different grains, a relation curve between a landscape index and grain in the study area was obtained by using landscape index and grain values as the horizontal axis and vertical axis, respectively. The dynamic curve associated with a landscape index over time in the study area was gained by using time point and landscape index values as the horizontal axis and vertical axis, respectively [52].

#### 2.3.4. Loss of Information under Different Landscape Scales

Loss of information of landscape area under different spatial grains was calculated to select an optimal spatial grain for exploring landscape pattern. In the study area, the higher difference index of landscape area ($S_{i}$) implies the higher loss of information caused by changes of spatial grain [53].

(1) $$\begin{array}{c} L_{i}=\frac{\left(A_{i}-A_{bi}\right)}{A_{bi}}\times 100\%\\ S_{i}=\sqrt{\frac{\sum_{i=1}^{n}{L_{i}}^{2}}{n}} \end{array}$$

where $A_{i}$ is the grid area of landscape type i. $A_{bi}$ is the basic vector of scale changes of one landscape type. $L_{i}$ is the loss differential ratio of landscape area. n is the number of landscape types in the study area.

## 3. Results and Analysis

### 3.1. Grain Effect of Landscape Pattern in Yancheng Coastal Wetland

The CV of landscape indexes on the landscape level and class level in the study area was calculated. It was found that different landscape indexes had significantly different responses to grain size changes. Among the 15 landscape-level indexes (Table 3), perimeter-area ratio (PARA) presented, the extremely strong response sensitivity to grains and CV was increased by 3.06, reaching the maximum in 2008. Number of patches (NP), Patch density (PD), and Mean patch area (MPS) showed strong response sensitivity to the grains. Specifically, NP and PD made strong responses to grain changes in 2000, reaching the extremely strong response sensitivity. However, the response sensitivities of NP, PD, and MPS declined gradually overall. Edge density (ED), Landscape shape index (LSI), and Contiguity index (CONTIG) showed moderate response sensitivity to grains. Their response sensitivity to the grains declined gradually in the study area. Largest patch index (LPI), Mean shape index (MSI), Fractal dimension index (FRAC), Shannon’s diversity index (SHDI), Shannon’s evenness index (SHEI), and Aggregation index (AI) showed low response sensitivity to the grains, while Patch richness (PR) showed response insensitivity to the grains. Per class-level landscape indexes (Table 4), CV of 12 class -level landscape indexes in the study area in 2017 showed that three landscape indexes (NP, PD, and MPS) presented extremely strong response sensitivity to the grains. The CV of MPS was the highest among ten landscape types in the study area. AI showed strong response sensitivity to the grains. ED, CONTIG, PARA, LSI, and MSI showed moderate response sensitivity to the grains, while Total Area (CA), LPI, and FRAC were the least sensitive to the grain changes.

In short, ED and MPS, belonging to the area-edge index, and PARA and CONTIG, belonging to shape index, were strongly sensitive to the grains. Sensitivities of NP, PD, AI, Contagion index (CONTAG), and LSI, belonging to the aggregation index, were different to some extent. On both the class-level and landscape-level, NP and PD were sensitive to the grain changes, while AI and LSI were moderately sensitive to the grain changes. The sensitivity of class-level AI and LSI to the grains was stronger than that of landscape-level AI and LSI. At the landscape level, CONTAG was less sensitive to the grain changes. Among the diversity indexes, PR, SHDI, and SHEI were less sensitive to the grain changes. PR was insensitive to the grain changes.

**Figure 2:**
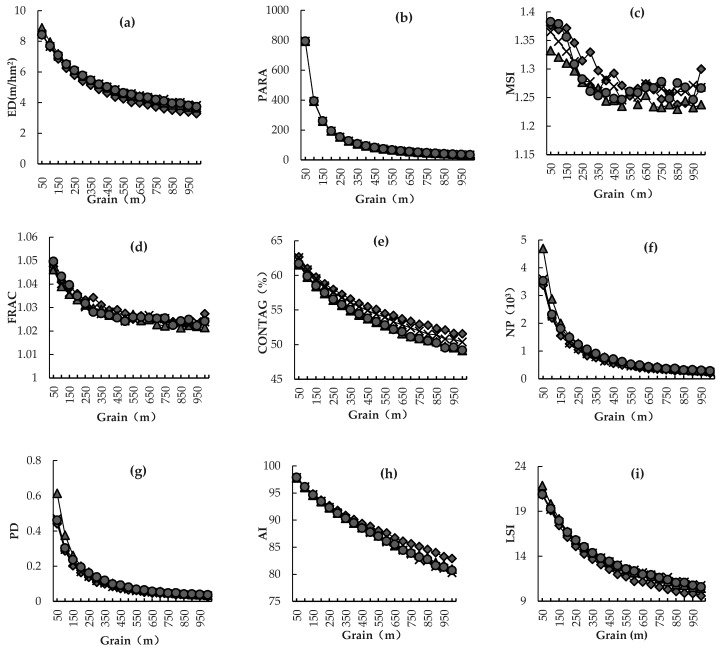

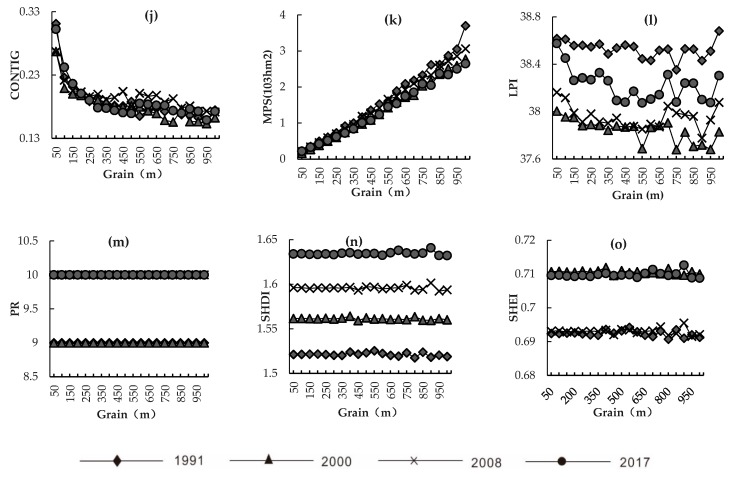
Relation curves between landscape-level landscape indexes and spatial grains. (**a**): ED, Edge density; (**b**): PARA, Perimeter-area ratio; (**c**): MSI, Mean shape index; (**d**): FRAC, Fractal dimension index ; (**e**): CONTAG, Contagion index; (**f**): NP, Number of patches; (**g**): PD, Patch density; (**h**): AI, Aggregation index; (**i**): LSI, Landscape shape index; (**j**): CONTIG, Contiguity index; (**k**): MPS, Mean patch area; (**l**): LPI, Largest patch index; (**m**): PR, Patch richness; (**n**): SHDI, Shannon’s diversity index; (**o**):SHEI, Shannon’s evenness index.

Different landscape indexes change differently with the increase of grain size. Relation curves between landscape-level landscape indexes and grains are shown in Figure 2, which covers six types of landscape indexes. The first type of landscape indexes included ED, CONTAG, and AI (Figure 2a,e,h). All indexes decreased slowly with the increase of grain size, which was manifested by smoothing relation curves between these landscape indexes and grains. In general, the difference of landscape indexes was slowly reduced at different grain levels. These landscape indexes dropped dramatically in the grain size range of 50–350 m, but they changed slightly in the range of 400–1000 m. The second type of landscape indexes included PARA, NP, PD, and LSI (Figure 2b,f,g,i). With the increase of grain size, these landscape indexes decreased quickly and then decreased slowly. They achieved a great reduction in the grain size range of 50–100 m, but they were decreased slightly in the range of 100–250 m, and even more slightly in the range of 250–1000 m. The all relation curves between these landscape indexes and grains became smoother after 250 m. The third type of landscape index was MPS (Figure 2k), which increased slightly in the grain size range of 50–150 m, but it increased quickly in the range of 150–1000 m. The fourth type of landscape indexes included MSI, FRAC, and CONTIG (Figure 2c,d,j), which decreased in a fluctuating manner with the increase of grain size. They decreased sharply in the grain size range of 50–150 m, but decreased slightly in the range of 150–1000 m. The fifth type was LPI, which fluctuated with the increase of grain size. LPI (Figure 2l) dropped significantly in the grain size range of 50–150 m in 2017, but it increased again and then decreased in the range of 150–300 m. It fluctuated in the grain size range of 300–1000 m. The sixth type of landscape indexes mainly reflected diversity of landscape, including PR, SHDI, and SHEI (Figure 2m–o). These three indexes were basically stable with the grain size.

**Figure 3:**
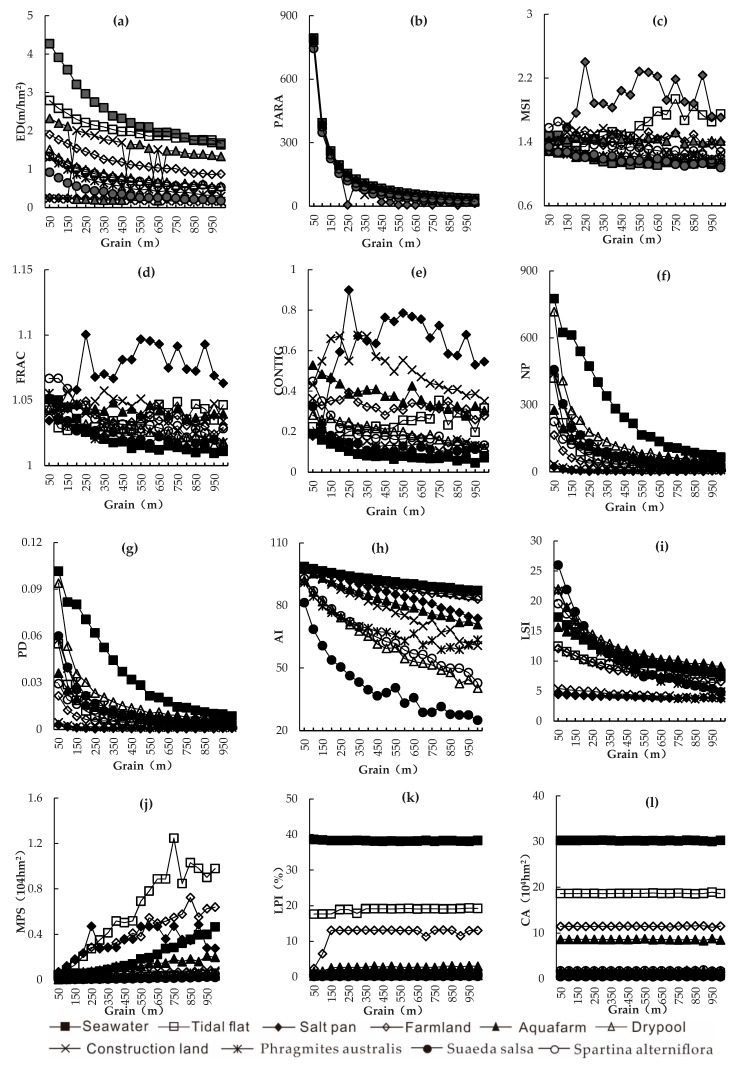
Relation curves between class-level landscape indexes and spatial grains. (**a**): ED, Edge density; (**b**): PARA, Perimeter-area ratio; (**c**): MSI, Mean shape index; (**d**): FRAC, Fractal dimension index ; (**e**): CONTIG, Contiguity index; (**f**): NP, Number of patches; (**g**): PD, Patch density; (**h**): AI, Aggregation index; (**i**): LSI, Landscape shape index; (**j**): MPS, Mean patch area; (**k**): LPI, Largest patch index; (**l**): CA, Total Area.

Variation trends of class-level landscape indexes with the grain size were analyzed based on the six types of landscape indexes (Figure 3). With respect to the relation curve between ED (Figure 3a) and grains, seawater decreased significantly and then decreased slowly, while tidal flat, farmland, dry pond, *Phragmites australis*, *Suaeda salsa*, and *Spartina alterniflora* decreased slowly. Construction land and aquafarm fluctuated in opposite directions and presented ladder-like backward movement. ED of salt pan remained basically stable with changes of grain size. PARA (Figure 3b) of salt pan reached the minimum at the grain of 250 m. However, PARA of all other nine landscape types decreased sharply and then slowly. Specifically, the PARA decreased mostly in the grain size range of 50–100 m, and it decreased continuously and slowly in the range of 100–200 m. Subsequently, PARA became increasingly stable. The relation curves of MSI, FRAC, and CONTIG (Figure 3c–e) with grains were similar. MSI, FRAC, and CONTIG of all ten landscape types fluctuated with changes of grain size. MIS of salt pan and tidal flat fluctuated the most. FRAC of salt pan changed the most. CONTIG of all the nine landscape types except seawater fluctuated significantly. Variation curves of NP, PD, and LSI with the grain size were similar. The variation curves of NP, PD, and LSI (Figure 3f,g,i) of salt pan and construction with the grains were smooth, but curves of the other eight landscape types decreased sharply and then slowly. The maximum reduction was observed in the grain size range of 50–100 m. AI and MPS (Figure 3h,j) presented opposite changes with the increase of grain size. In *Suaeda salsa*, AI decreased significantly and then became stable with changes of grain size. However, the relation curves between AI and grains in other landscape types decreased slowly. MPS of tidal flat, farmland, salt pan, seawater, and aquafarm increased sharply with the increase of grain size, but the MPS of other landscape types presented small growth. Variation curves of LPI and CA (Figure 3k,l) with the grains were similar, which were stable in a straight line. LPI of farmland soared up in the grain size range of 50–150 m, but it remained stable with changes of grain size, as with other landscape types.

### 3.2. Response of Landscape Grain Effect to Landscape Changes in Yancheng Coastal Wetland

As an important component of the world’s wetlands, Yancheng Coastal Wetland has witnessed great changes in landscape patterns as a result of rapid marine economic development, continuous progress in tideland reclamation, and continuous expansion of culture ponds in Jiangsu Province, China. The landscape indexes presented different characteristics of grain effect at different time points, which might also respond to landscape change.

**Figure 4:**
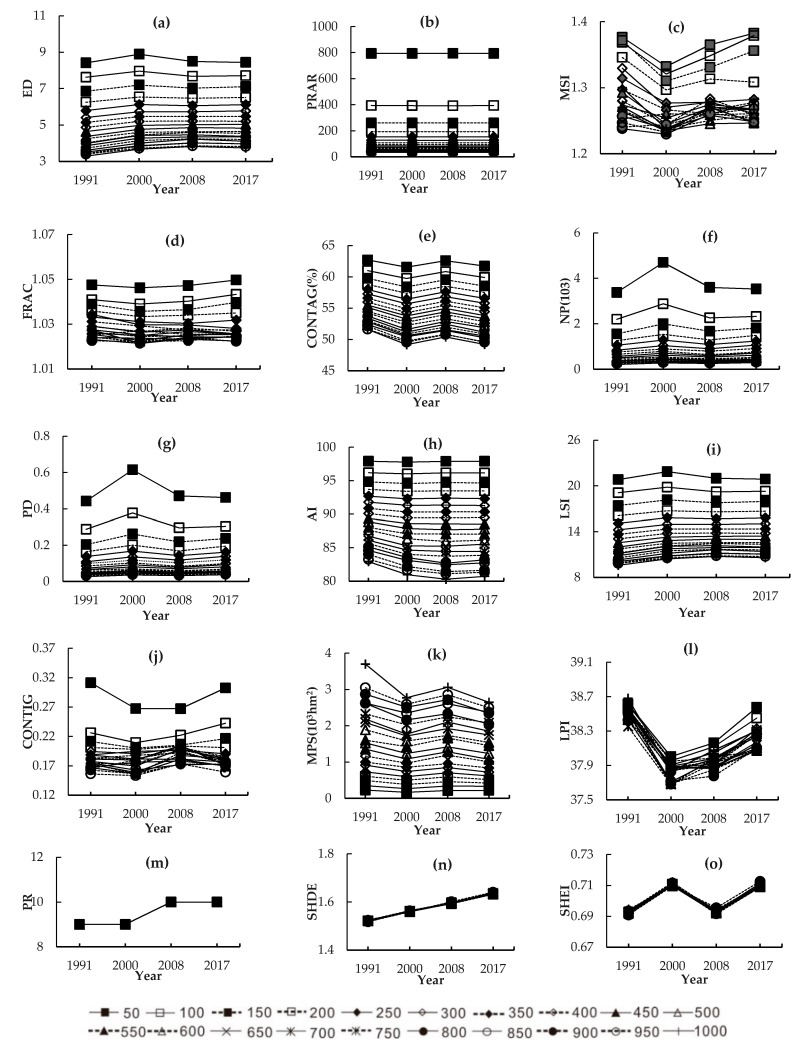
Relation curves between spatial grains at the landscape level over time. (**a**): ED, Edge density; (**b**): PARA, Perimeter-area ratio; (**c**): MSI, Mean shape index; (**d**): FRAC, Fractal dimension index ; (**e**): CONTAG, Contagion index; (**f**): NP, Number of patches; (**g**): PD, Patch density; (**h**): AI, Aggregation index; (**i**): LSI, Landscape shape index; (**j**): CONTIG, Contiguity index; (**k**): MPS, Mean patch area; (**l**): LPI, Largest patch index; (**m**): PR, Patch richness; (**n**): SHDI, Shannon’s diversity index; (**o**): SHEI, Shannon’s evenness index.

The landscape indexes can be divided into four types according to the variation curves of grains at different time points (Figure 4). The first type of landscape index increased slightly in a fluctuating manner as time went on and the variation trends with grains were generally stable. Moreover, these landscape indexes were proportional to grains in different years. This type of landscape index included ED, FRAC, and LSI (Figure 4a,d,i), which reflected the effects of landscape dynamic changes on landscape area and morphology in the study area. The landscape morphology became increasingly complicated. The second type of landscape index increased in a fluctuating manner as time went on. They kept similar variation trends with grains in different years and fluctuated greatly when the grain size ranged from 50 m to 200 m, especially at 50 m and 100 m. This type of landscape index included MSI, NP, PD, CONTIG, and LPI (Figure 4c,f,g,j,l). Most of the landscape indexes in this type belong to the Aggregation index, indicating significant impacts of landscape pattern changes on the aggregation degree of landscapes in the study area. The number and density of patches increased, while the proximity and maximum patch index decreased. The third type of landscape index decreased over time and their relation curves with grains showed a similar trend. The landscape indexes in this type included PARA, CONTAG, AI, and MPS (Figure 4b,e,h,k). The relation curve between MPS and grains was the opposite of the relation curves of PARA, CONTAG, and AI with gains. MPS reached the peak when the grain size was 1000 m and it was positively related with grains. On the contrary, PARA, CONTAG, and AI reached the maximum when the grain was 50 m and these indexes were negatively correlated with grains. In the study area, the average patch area decreased, indicating an increase of fragmentation of the landscape patch and a decrease of the dominance of major landscapes. AI and CONTAG decreased, indicating that the connection of different landscape patches was broken and the landscape integrity was weakened. The fourth type of landscape index increased as time went on, but remained stable regardless of changes of grains. This type of landscape index included PR, SHDI, and SHEI (Figure 4m–o), which increased independently. The PR of different landscape types was different, but it was unrelated with grains. SHDI increased continuously and kept the same growth trend with the increase of grains, indicating the uniform distribution of different landscape types, and the dominance of landscape decreased in the study area. On the contrary, SHEI approached 1 continuously, indicating that the landscape diversity was increased and landscape distribution was more even in the region.

### 3.3. Responses of Landscape Grain Effect of Natural and Artificial Wetlands to Landscape Changes

In the study area, landscapes could be divided into ten types. The study area could be divided into natural and artificial wetlands depending on the impacts and disturbances of human activities. The former is less influenced by human activities, which has extensive distributions of tidal flat, *Phragmites australis*, *Suaeda salsa*, and *Spartina alterniflora*. The latter expands toward the sea area, which is mainly manifested as reclamation for aquafarm, dry pond, salt pan, and farmland. There are dense reclamation regions in coastal areas in Xiangshui, Binghai, and Sheyang Counties in Yancheng. Considering the complexity of land types, responses of the landscape grain effect to landscape changes were investigated by using natural and artificial wetlands. Representative landscape indexes FRAC, MPS, and AI were chosen to reflect changes of the area-edge, shape, and degree of aggregation in natural and artificial wetlands at different time points (Table 5).

FRAC can reflect complicated conditions of different landscapes or patch shapes, with a value between 1 and 2. When FRAC approaches 1, the patch morphology of landscape types tends to be small and regular. When FRAC approaches 2, the plaque morphology is irregular and more complicated. Generally, the upper limit of FRAC is 1.5. There is a small difference in FRAC between natural and artificial wetlands in the study area. It was 1.03 in 1991 and 2000, and 1.04 in 2008 and 2017, indicating small morphological changes of landscape patches in natural and artificial wetlands with the change of grains. The landscape patch tended to be regularized. CV was equal and relatively low at the early stage. However, the CV of FRAC in the artificial wetland was higher than that in the natural wetland, indicating that FRAC of artificial wetland had a small response to grain size changes.

MPS reflects the changes of patch area of different landscape types. In the study area, the MPS of both natural and artificial wetlands was decreased by 426.49 hm^2^ and 443.37 hm^2^, respectively. This reflects that patches of natural and artificial wetlands tended to be fragmented and a large patch was cut apart. The average patch area in the artificial wetland decreased more than that in the natural wetland. However, MPS tended to increase during 2008–2017, which was attributed to the implementation of ecological protection policy against sea reclamation. For example, the core zone, buffer zone, and test zone restrict landscape development to a large extent and play an important role in landscape protection. The CV of the MPS of natural and artificial wetlands was extremely strongly sensitive to the increase of the grain size. CV increased firstly, then decreased, and finally increased again, overall gradually declining. The response of natural wetland to grain size changes was higher than that of artificial wetland.

AI reflects the aggregation in patches of landscape types. The higher value of AI reflects the better connection of patches in landscapes in the region and a patch is larger, simpler, and more regular. Lower value of AI implies the higher fragmentation and irregularity of patches in landscapes, accompanied with a scattered distribution of small patches. The AI of natural and artificial wetlands increased continuously, indicating the improving connectivity of landscapes and increased aggregation of patches in the study area. In particular, the prohibition of development activities in the core zone and differential protections in the buffer zone and test zone achieved a certain effect. There were dense distributions of *Phragmites australis*, *Spartina alterniflora*, and *Suaeda salsa* in the tidal flats in the coastal wetland, forming a natural buffer zone. Due to sea reclamation and farming reclamation in the artificial wetland, patches became more aggregated. The AI in natural wetlands was generally higher than that in artificial wetlands. Both natural and artificial wetlands presented moderate response sensitivity to the grain size changes and the response degree decreased. Response of natural wetlands to the grain changes was stronger than that of artificial wetlands.

### 3.4. Selection of an Optimal Grain Size for Landscape Pattern Analysis in the Study Area

The landscape area difference index in the study area under different grains in 1991 and 2017 was calculated according to Equation (1). On this basis, the degree of loss of information of the landscape under different grains was gained. When the grain size increased from 50 m to 1000 m continuously, relevant attributes, such as position of landscape patch, patch in adjacent region, perimeter, and area, changed during resampling of landscape grid data. This also caused differences of landscape patterns under different grain effects. Hence, the variation in the accuracy of landscape areas under different grains was analyzed, aiming to identify the optimal grain size that corresponds to the minimum loss of information of landscape patches.

**Figure 5:**
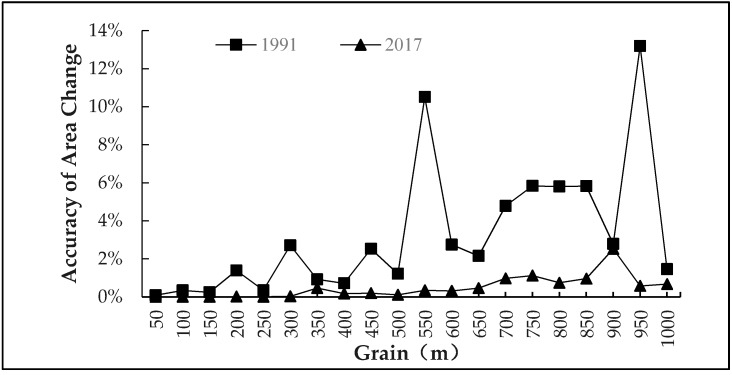
The accuracy of landscape area variation under different grains in 1991 and 2017.

Landscape data of the study area at the beginning stage and end stage of the study period were calculated, thus enabling us to determine the variation accuracy curve of landscape area under different grains (Figure 5). It was discovered that with the increase of grain size, the area variation accuracy fluctuated. The area variation accuracy had two peak points in 1991, which were at 550 m and 950 m. These two points had the highest loss of information. The area variation accuracy was small in the grain size range of 50–150 m. The area variation accuracy curve in 2017 was relatively smooth. The maximum loss of information of the landscape was achieved at 900 m, but there is a small loss of information in the range of 50–250 m. The area variation accuracy curves at the two stages demonstrate that the optimal grain size for analysis was between 50 and 250 m. Based on comparison of area variation accuracies at 50 m, 100 m, 150 m, 200 m, and 250 m, as well as previous studies on the grain effect of landscape indexes, the optimal grain size to analyze the landscape pattern in the study area was 50 m. This optimal grain size not only could improve computation accuracy, but also assured the integrity of landscape information.

## 4. Discussion

### 4.1. Response Sensitivity of Landscape Indexes to Grains

In this study, we systematically analyzed changes of various landscape indexes at multiple grain levels, including multiple indexes at landscape level and class level, so as to make the analyzed landscape more generally representative. A total of 27 common landscape indexes were chosen in the present study, including 12 class-level indexes and 15 landscape-level indexes. Among them, seven landscape indexes showed extremely strong response sensitivity to grain size changes. Five landscape indexes presented strong response sensitivity to the grain changes. Six landscape indexes presented moderate response sensitivity to the grain changes. Seven landscape indexes presented low response sensitivity to the grain changes. Only one landscape index (PR) was insensitive to the grain changes. Our conclusions were similar with the conclusions derived by Chen et al. [52] and Teng et al. [45]. In summary, area-edge indexes and shape indexes were more sensitive to the grain changes. Aggregation indexes (NP, PD, AI, CONTAG, and LSI) had different responses to the grain changes. Diversity indexes (PR, SHDI, and SHEI) were less sensitivity to the grain changes. Moreover, PR was insensitive to the grain changes. During the transition from 50 m to 2000 m, the landscape type patches also began to change. As the landscape grain size increased, the grid of one or several landscape types began to expand, and the patch edge and shape of the landscape type changed, directly acting on the perimeter of the landscape patch and the average patch area. In addition, the aggregation index, such as the number, density, and aggregation of patches, also changed accordingly. The three indices of diversity were related to each other. Because the type of landscape patches was relatively fixed, the PR value was mainly nine or ten. The increase of SHDI and the proximity of SHEI to 1 indicated that there was no obvious dominant type, and the patch type was uniform in the landscape. The trend distribution and landscape heterogeneity increased [54]. Therefore, the particle grain effect relationship based on these landscape analyses also had a certain universality and commonality.

### 4.2. Changes of the Different Grain Size Effects of Landscape Indices

Relation curves between landscape indexes and grains could be divided into six types. The first type was slow declining, including ED, CONTAG, and AI (Figure 2a,e). The relation curves of this type were smooth and had a sharp reduction when the grain size increased from 50 m to 350 m. The second type decreased quickly and then slowly, including PARA, NP, PD, and LSI (Figure 2b,f,g,i). These curves dropped mostly in the grain size range of 50–100 m and became smoother after 250 m. The third type increased slightly in the range of 50–150 m, but it increased quickly in the range of 150–1000 m. This type included MPS (Figure 2k). The fourth type was declining in a fluctuating manner and the curves were not smooth, including MSI, FRAC, and CONTIG (Figure 2c,d,j). The fifth type was fluctuating curve, which included LPI (Figure 2l). The sixth type was stable, without an evident rising or declining trend. This type included PR, SHDI, and SHEI (Figure 2m–o). Research results in this study were slightly different from those in previous studies by Chen et al. [52]. This might be caused by the different grain size in this study area. Chen et al. [52] divided the grain size in the study area into 30 m, 50 m, and 100 m. However, the grain size in this study was divided into 50 m, 100 m, …, 950 m, and 1000 m, respectively. Hence, the response of landscape indexes to the grain changes was closely related with sampling grid density. Compared with previous studies, in this study, we expanded the scale range of the landscape grain analysis, which meant we could analyze the response of landscape index to the change of grains more objectively and accurately. It could provide a reference for index selection, result interpretation, and spatial scale generalization in landscape pattern analysis.

### 4.3. Trends and Responses of Landscape Indices with Spatial Grains

Variation curves of landscape grains could be divided into four types. The first type increased in a fluctuation manner. The variation trend of this type of landscape index (ED, FRAC, and LSI) (Figure 4a,d,i) was basically consistent, indicating that the landscape morphology became increasingly complicated. The second type presented fluctuating curves, including MSI, NP, PD, CONTIG, and LPI (Figure 4c,f,g,j,l). The relation curves of MSI, NP, PD, CONTIG, and LPI with grains were similar and fluctuated mostly when the grains ranged between 50 m and 100 m. This was manifested by the increased number and density of patches but decreased proximity and maximum patch index. The third type was monotonous declining curves, including PARA, CONTAG, AI, and MPS (Figure 4b,e,h,k). The average patch area decreased, but the fragmentation of the landscape patch increased, due the reduction of the major landscape in the study area. Connections of different landscape patches were broken and the landscape integrity was weakened. The fourth type was monotonous rising curves, mainly including PR, SHDI, and SHEI (Figure 4m–o). These landscape indexes were stable under different grain sizes. There is a uniform distribution of different landscape types in the study area. Specifically, the leading position and dominance of a landscape declined, while landscape diversity increased. To sum up, shape, aggregation index, and area-edge indexes had stronger responses to the grain size changes, while the diversity indexes remained basically the same when the grains increased from 50 m to 1000 m. During 1991–2017, landscapes in the study area tended to be fragmented and complicated under intensive development, indicating the weakening internal connectivity, shrinking dominant landscape areas, and aggregated distribution of small patches.

From the perspective of different grain effect changes of natural and artificial wetland landscape indexes, FRAC of natural and artificial wetlands in the study area changed slightly from 1991 to 2017. MPS decreased firstly and then increased, while AI increased. Generally, the natural wetland was more sensitive to grain size than the artificial wetland. The shapes of the landscape patch in the natural and artificial wetlands changed slightly, but patch tended to be fragmented. Moreover, the MPS of the artificial wetland decreased more than that of the natural wetland. Subsequently, MPS tended to increase and AI increased, which improved connectivity of landscapes in the study area. The increased AI was attributed to enhanced protection efforts in the study area, such as the foundation of the core zone, buffer zone, and test zone and various coastal wetland parks as well. Such efforts have actively declared the natural heritage of coastal wetlands, and introduced laws and regulations to protect wetlands, such as “the Yancheng Yellow Sea Wetland Protection Regulations” (set by the Standing Committee of Yancheng Municipal People’s Congress) and “Jiangsu Province Wetland Protection Master Plan (2006–2030)” (set by the Forestry Bureau of Jiangsu Province, China), etc., have played a key role in the protection of Yancheng’s natural wetlands. The connectivity of the landscape was increased in the study region. Influenced by sea reclamation and farming reclamation, patches of aquafarm, salt pan, farmland, and dry ponds tended to aggregate in the artificial wetland. By a comparative analysis of natural and artificial wetland areas, it was found that the natural wetland area shrank due to human disturbances. The artificial wetland area tended to expand quickly. The MPS of the landscape in the study area declined and patch became increasingly fragmented. This confirms research conclusions by Zhang et al. [53].

### 4.4. The Optimal Scale of Spatial Grain in Landsacpe Pattern Analysis

The area variation accuracy curves at two stages in 1991 and 2017 demonstrated that an optimal landscape grain size in the study area ranged between 50–250 m. Landscape indexes at 50 m had the strongest responses to the grain changes. The loss of information of landscape reached the minimum at 50 m grain size. Therefore, the optimal landscape grain size to study the landscape pattern in the study area was determined to be 50 m. This is similar with the research conclusion derived by Chen et al. [52] (30–60 m) and Wu et al. [33], but it is significantly different from the value (200 m) obtained by Sun et al. [51]. This is because the Sun et al. [48] only chose two landscape indexes (FRAC and PARA), but this study involved 27 landscape indexes. The optimal grain size was determined based on the loss of information of the landscape under different grains and it reflected the accuracy and integrity of the research better.

Studying the effect of grain size is a basic premise of landscape analysis [17,42,55,56]. The landscape pattern in the study area was significantly different under different grain sizes, which might further influence the acquisition of landscape information [1,57]. Hence, choosing an optimal grain size was of important significance to study the landscape pattern in the study area [37,53]. An optimal grain size can not only acquire landscape information, but also simplify the landscape data and avoid data redundancy. In this study, the sensitivity of landscape indexes to grain changes was analyzed. The grain effect is not be limited to analysis. Therefore, the grain effect should be analyzed more in ecosystem studies, thus strengthening grain response studies [58,59].

## 5. Conclusions

In this study, based on landscape data acquired in 1991, 2000, 2008, and 2017, the landscape grain effect and response of landscape grains (50–1000 m, with an interval of 50 m) to landscape changes in the Yancheng Coastal Wetland were analyzed. The optimal landscape grain size chosen in this study and several conclusions are summarized as follows:

(1) The response sensitivities of different landscape indexes to different grain sizes varied greatly. Among the 27 landscape indexes selected in this study, four were extremely sensitive to the spatial grain change, four were highly sensitive, ten were moderately sensitive, eight were low sensitive, and one was not sensitive. At the class level, from the selected coefficient of variation of 12 landscape indexes, it could be seen that three landscape indexes were extremely sensitive to the grain difference, one was highly sensitive, six were moderately sensitive, and two were of low sensitivity.

(2) The landscape index showed diversity characteristics as the spatial grain size increases. Generally, they could be divided into six types. The first type was characterized by slow decline, and included ED, CONTAG, AI. The second type decreased quickly and then slowly, including PARA, NP, PD, and LSI. The third type was monotonically rising, including MPS. The fourth type was declining in a fluctuating manner, including the MSI, FRAC, and CONTIG indexes. The fifth type was presented a fluctuating curve, which included LPI. The sixth type was stable, including PR, SHDI, SHEI. With respect to landscape types, the ED of seawater declined quickly at first and then slowly. The ED of tidal flat, farmland, dry pond, *Phragmites australis*, *Spartina alterniflora*, and *Suaeda salsa* decreased slowly. The ED of construction land and aquafarm fluctuated, while the ED of salt pan was stable. The PARA of all landscape types decreased quickly and then slowly, except that the PARA of salt pan reached the minimum at 250 m. The MSI, FRAC, and CONTIG curves changed similarly, indicating that the landscape types were fluctuating. The NP, PD, and LSI curves were similar. The NP, PD, and LSI curves of salt pan and construction land were smooth, but the NP, PD, and LSI curves of the other eight landscape types decreased quickly and then slowly. AI and MPS changed oppositely with grain size. LPI and CA curves were basically straight across different grains.

(3) The response of the landscape grain effect to the process of landscape change was significant. The response results were mainly divided into four types. The first type went up in a fluctuation manner, including ED, FRAC, and LSI. The second type presented fluctuating curves, mainly including MSI, NP, PD, CONTIG, and LPI. The third type presented monotonously declining curves, including PARA, CONTAG, AI, and MPS. The fourth type presented monotonously rising curves, including PR, SHDI, and SHEI. The response of the landscape grain effect to a landscape change process was different between natural wetland and artificial wetland. Overall, natural wetlands were more sensitive to the grain effect than artificial wetlands.

(4) The variation of the grain effect of different landscapes was studied, and the accuracy of landscape area change under different grains was analyzed. Finally, the optimal analysis grain size for studying Yancheng Coastal Wetland landscape patterns was determined as 50 m.

In this study, we analyzed grain characteristics in the process of landscape change, selected the optimal spatial grain scale, and effectively eliminated redundant information. The conclusions derived from this study are conducive to comparisons of landscape pattern characteristics and the interpretation of analysis results. Thus, this study can aid the analysis of ecosystem functions and lay the foundation for ecological risk assessment and ecosystem value estimation. In addition, further research on the topic addressed in this study is needed in the future.

## Figures and Tables

**Table 1:** Satellite remote sensing data.

Satellite	Sensor	Path/Row	Date	Satellite	Sensors	Path/Row	Date
Landsat 5	TM	120/36	1991-11-19	Landsat5	TM	119/37	1991-11-28
Landsat 5	TM	120/36	2000-12-13	Landsat5	TM	119/37	2000-12-06
Landsat 5	TM	120/36	2008-12-19	Landsat5	TM	119/37	2009-01-13
Landsat 8	OLI	120/36	2017-12-01	Landsat8	OLI	119/37	2017-12-10

**Table 2:** Landscape pattern indexes.

Landscape Pattern Index Type	Landscape Pattern Indexes	Index Name	Class Level	Landscape Level
Area -margin index	Total area	CA	√	
	Largest patch index	LPI	√	√
	Edge density	ED	√	√
	Mean patch area	MPS	√	√
Shape index	Perimeter-area ratio	PARA	√	√
	Mean shape index	MSI	√	√
	Fractal dimension index	FRAC	√	√
	Contiguity index	CONTIG	√	√
Aggregation index	Number of patches	NP	√	√
	Patch density	PD	√	√
	Aggregation index	AI	√	√
	Contagion index	CONTAG		√
	Landscape shape index	LSI	√	√
Diversity index	Patch richness	PR		√
	Shannon’s diversity index	SHDI		√
	Shannon’s evenness index	SHEI		√

**Table 3:** Change characteristics of landscape-level landscape indexes with grains (%).

Year	NP	PD	LPI	ED	LSI	MSI	FRAC	PARA	CONTIG	CONTAG	PR	SHDI	SHEI	AI	MPS
1991	96.64	96.64	0.19	29.99	24.46	3.42	1.64	125.74	17.45	5.66	0	0.13	0.13	4.83	58.27
2000	108.00	107.97	0.25	28.46	23.54	2.52	1.64	129.39	14.33	6.65	0	0.08	0.08	5.68	59.85
2008	99.60	99.59	0.25	25.47	21.09	2.55	1.62	129.71	10.79	6.49	0	0.13	0.13	6.01	57.29
2017	90.70	90.67	0.36	26.10	21.60	3.31	1.72	128.80	17.20	6.78	0	0.12	0.12	5.86	57.21

**Table 4:** Change characteristics of class-level landscape indexes with grains in 2017 (%).

Landscape types	CA	NP	PD	LPI	ED	LSI	MSI	FRAC	PARA	CONTIG	AI	MPS
Seawater	0.23	78.71	78.70	0.36	31.66	25.43	9.93	1.31	40.86	3.71	70.52	128.72
Tidal flat	0.39	143.26	143.08	3.13	14.75	14.85	12.88	0.73	21.63	4.87	58.15	128.90
Salt pan	1.19	83.07	81.99	2.72	5.01	5.14	15.57	1.83	28.32	8.83	37.48	180.22
Farmland	0.64	87.08	86.85	22.76	25.86	20.52	4.10	0.71	9.36	5.01	45.07	131.33
Aquafarm	1.08	65.49	65.45	15.75	17.56	17.06	2.37	0.39	17.22	9.81	42.06	132.26
Dry pond	2.04	105.24	105.16	13.76	32.32	31.69	3.10	0.61	24.49	24.38	53.49	139.18
Construction land	2.97	48.77	49.45	2.80	12.50	11.65	8.29	0.83	20.73	14.28	23.52	141.79
*Phragmites australis*	2.58	113.13	113.00	4.32	47.74	48.27	4.20	0.90	38.07	12.78	68.56	139.43
*Suaeda salsa*	3.90	115.41	115.31	23.09	53.98	55.32	5.64	0.90	42.58	36.75	66.77	155.69
*Spartina alterniflora*	3.23	62.86	62.76	12.62	31.61	31.69	9.51	1.33	41.23	21.62	42.85	141.78

**Table 5:** Change characteristics of landscape indexes in natural and artificial wetlands under different spatial grains during 1991−2017.

Year	Fractal Dimension Index (FRAC)				Mean Patch Area (MPS)				Aggregation Index (AI)			
	Natural Wetland		Artificial Wetland		Natural Wetland		Artificial Wetland		Natural Wetland		Artificial Wetland	
	Mean	CV/%	Mean	CV/%	Mean	CV/%	Mean	CV/%	Mean	CV/%	Mean	CV/%
1991	1.03	1.31	1.03	1.31	2251.87	131.71	2289.06	130.54	73.55	30.66	73.72	29.78
2000	1.03	1.45	1.03	1.45	2297.15	140.43	2253.48	138.74	74.40	26.39	74.31	26.01
2008	1.04	1.40	1.04	1.40	1634.47	119.07	1659.54	117.97	75.79	23.12	75.84	22.74
2017	1.04	1.71	1.04	1.72	1825.38	130.85	1845.69	130.08	75.47	24.44	75.29	24.46
